# Bifidobacterial Dialogue With Its Human Host and Consequent Modulation of the Immune System

**DOI:** 10.3389/fimmu.2019.02348

**Published:** 2019-10-01

**Authors:** Giulia Alessandri, Maria Cristina Ossiprandi, John MacSharry, Douwe van Sinderen, Marco Ventura

**Affiliations:** ^1^Department of Veterinary Medical Science, University of Parma, Parma, Italy; ^2^Microbiome Research Hub, University of Parma, Parma, Italy; ^3^APC Microbiome Institute and School of Microbiology, Bioscience Institute, National University of Ireland, Cork, Ireland; ^4^Laboratory of Probiogenomics, Department of Chemistry, Life Sciences, and Environmental Sustainability, University of Parma, Parma, Italy

**Keywords:** bifidobacteria, immune system, immunomodulation, host interaction, probiotics

## Abstract

Since bifidobacteria are among the pioneering colonizers of the human infant gut, their interaction with their host is believed to start soon following birth. Several members of the *Bifidobacterium* genus are purported to exert various health-promoting effects at local and systemic levels, e.g., limiting pathogen colonization/invasion, influencing gut homeostasis, and influencing the immune system through changes in innate and/or adaptive immune responses. This has promoted extensive research efforts to shed light on the precise mechanisms by which bifidobacteria are able to stimulate and interact with the host immune system. These studies uncovered a variety of secreted or surface-associated molecules that act as essential mediators for the establishment of a bifidobacteria-host immune system dialogue, and that allow interactions with mucosa-associated immune cells. Additionally, the by-products generated from bifidobacterial carbohydrate metabolism act as vectors that directly and indirectly trigger the host immune response, the latter by stimulating growth of other commensal microorganisms such as propionate- or butyrate-producing bacteria. This review is aimed to provide a comprehensive overview on the wide variety of strategies employed by bifidobacteria to engage with the host immune system.

## Introduction

The gastrointestinal tract (GIT) of humans, and by extension of mammals, harbors an extremely dense, and complex community of microorganisms. Until recently, this microbial ensemble, collectively forming the so-called gut microbiota, was estimated to exceed the total number of the host cells by about 10-fold (1, 2). However, this number has been recently revised to a 1:1 ratio (3, 4). Despite this downward revision, the gut microbiota remains the most complex of the human-associated microbial communities and rendering the intestine one of the most intricate bacterial habitats in the biosphere. Millions of years of co-evolution between the mammalian host and its intestinal microbial ecosystem has led to the establishment of various trophic interactions, including a mutualistic relationship in which the host ensures nourishment and a suitable environment for the microbial community, while the microbiota, in turn, provides the host with multiple physiological and metabolic functions (5, 6). Beyond the provision of nutrition for enterocytes and degradation of non-digestible food compounds, the gut microbiota is also involved in a continuous dialogue with the immune system promoting the maintenance of the intestinal barrier, influencing bowel homeostasis and functionality, stimulating optimal immune responses, and providing protection against pathogen colonization (6, 7). Functional and physiological alterations of the intestinal barrier are generally associated with damage of the barrier itself, leading to a broad range of both intestinal and systemic diseases, including inflammatory bowel diseases (IBD), colorectal cancer, or celiac disease (8, 9). Among the multitude of gut bacteria that have been shown to positively influence the immune system, many studies have focused their interest on bifidobacteria as commensal microorganisms that are able to stimulate and modulate specific pathways, influencing both innate and adaptive host immune responses (10, 11). However, current knowledge on the molecular mechanisms that are responsible for the cross talk between bifidobacteria and the host immune system remain superficial and incomplete.

In this review, we will discuss current knowledge on immune-modulatory activities exerted by bifidobacteria in the intestinal environment. Particularly, we will detail how specific bifidobacterial extracellular structures (are believed to) modulate the host immune response. Furthermore, we will discuss certain interactions between bifidobacteria and other intestinal microbes affect the host immune system.

## Taxonomic and Ecological Diversity of the *Bifidobacterium* Genus

Bifidobacteria are Gram-positive, anaerobic, non-motile, non-sporulating, saccharolytic, and Y-shaped microorganisms with a high G + C DNA content. They were described for the first time by Tissier in 1899, following their isolation from a fecal sample of a breast-fed infant. Bifidobacteria are classified as members of the *Bifidobacterium* genus, which forms a deep-branching lineage within the Actinobacteria phylum (12, 13). Currently, the *Bifidobacterium* genus encompasses 82 recognized taxa, representing 73 species and nine subspecies (14–17). A comparative genomic analysis based on 72 sequenced bifidobacterial type strains identified the presence of 261 *Bifidobacterium*-specific clusters of orthologous genes (COGs) shared by these genomes, collectively called the bifidobacterial core genome (18). The subsequent concatenation of the core gene protein sequences allowed the construction of a very robust phylogenetic tree highlighting the division of the *Bifidobacterium* genus into 10 phylogenetic groups, encompassing *Bifidobacterium adolescentis, Bifidobacterium boum, Bifidobacterium pullorum, Bifidobacterium asteroides, Bifidobacterium longum, Bifidobacterium psychraerophilum, Bifidobacterium bifidum, Bifidobacterium pseudolongum, Bifidobacterium bombi*, and *Bifidobacterium tissieri* group (18).

Bifidobacteria have been isolated from many ecological niches such as fermented milk (18), sewage (19), human blood (20), the oral cavity (21), and the gastrointestinal tract of mammals, birds, and insects (14, 16, 17, 22). Despite this apparent broad ecological distribution, the capability of bifidobacteria to adapt to different ecological niches is species-dependent. In this context, it has been demonstrated that certain bifidobacteria, such as *B. longum, B. adolescentis, B. pseudolongum*, and *B. bifidum*, display a cosmopolitan lifestyle, while other bifidobacterial taxa seem to be adapted to a gut ecosystem of a specific animal, for example *Bifidobacterium angulatum* in cows, *Bifidobacterium cuniculi* in rabbits and *Bifidobacterium gallinarum* in chickens (23, 24). The differential ecological success to adapt to various ecological niches very much relies on the genetic heritage of each bifidobacterial species. In this context, comparison of the genome sequences from the type strains of 47 *Bifidobacterium* (sub)species allowed the reconstruction of the bifidobacterial pan- and core-genome (25). The subsequent functional annotation of the core-genome by means of the EggNog database, indicated that a large part of the bifidobacterial core genes encode carbohydrate metabolism functions, thus suggesting that carbohydrate degradation plays an important role in bifidobacterial colonization of their ecological niches. Conversely, the pan-genome analysis revealed the identification of Truly Unique Genes (TUGs) corresponding to genes present in just one of the examined bifidobacterial genomes. Although the majority of TUGs cannot be attributed to a specific function, a portion of the TUGs were assigned to carbohydrate metabolism and transport functions, thus supporting the notion that the bifidobacterial gene repertoire for carbohydrate degradation plays an essential role in the differential ecological adaptation abilities among members of the *Bifidobacterium* genus (25). Similarly, *in silico* prediction of the bifidobacterial pan-secretome, i.e., the ensemble of genes encoding secreted proteins responsible for metabolism of nutrients, revealed the existence of species-specific bifidobacterial secretomes, which were predicted for the metabolism of particular glycans (26). These findings emphasize the correlation between the genetic repertoire of a *Bifidobacterium* species and their cosmopolitan or specialized lifestyle (26).

## Development of the Human Gut Microbiota and Associated Bifidobacterial Community

Despite the broad ecological distribution of bifidobacteria, certain human-associated bifidobacteria have gained particular importance as they are purported to exert health-promoting activities, especially in the context of the infant gut microbiota (27). For a long time, it was thought that the establishment of the human intestinal microbiota occurred immediately following birth. However, recent studies have disputed the dogma of a sterile *in utero* environment, providing evidence of a microbial presence in the placenta, amniotic fluid and umbilical cord in healthy full-term pregnancies, though these findings are very controversial (28–31). It is well-accepted that the very first microbial colonizers of the infant gut are represented by facultative anaerobes, including several members of the Enterobacteriaceae family that deplete the oxygen in the intestine (32). Following the removal of oxygen, the infant gut is extensively colonized by strictly anaerobic microorganisms, among which bifidobacteria are considered to be major players (33–35). However, the total load and diversity of bifidobacteria in the infant gut are strongly dependent on environmental factors such as mode of delivery (natural or C-section delivery), type of feeding (breast or formula milk), duration of gestation (full-term or premature birth), and antibiotic administration (29, 36–39). In this context, it has been demonstrated that naturally delivered and breast-fed infants generally possess a higher relative abundance and diversity in terms of bifidobacterial populations when compared to the gut microbiota of those infants born by Cesarean section and fed with formula milk (29, 40). Weaning and the concomitant transition to a solid and more varied diet cause an increase in the overall composition of the infant gut microbiota, reaching its peak in adult life. The exact age that marks the passage from an infant-like to an adult-like gut microbiota is not universally fixed, but it is generally thought to happen around 3 years (41, 42). Although at this age the gut microbiota seems to have reached the stability typical of an adult intestinal community, some microbial taxa are still far from reaching a steady state. Indeed, some differences in the microbial intestinal community are found between pre-adolescence and adulthood (43). In adult life, levels of bifidobacteria are reduced (compared to that during infancy), but remain stable over time until old age (43). The latter is characterized by a general decline in species diversity coupled with a decrease in bifidobacterial abundance (44). Similar to the changes in the overall microbial biodiversity and complexity that are observed at various ages, also the bifidobacterial community composition is subject to modifications. In this context, analyses of the bifidobacterial population by either culture or metagenomic approaches revealed stage-of-life-specific bifidobacterial strains. Indeed, *Bifidobacterium breve, B*. *bifidum*, and *B. longum* subsp. *infantis* are typically found in the gut of breastfed infants, while adult life is generally associated with higher abundance of *B. adolescentis* and *B. catenulatum* species (45, 46). Conversely, *B. longum* subsp. *longum* seems to have a ubiquitous distribution across the human lifespan (47).

## Immunomodulatory Effects Elicited by Bifidobacterial Extracellular Molecules Involved in Host-Microbe Interaction

**Figure 1:**
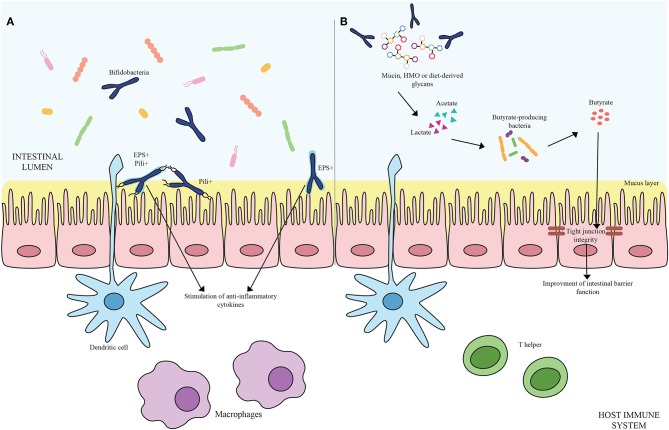
**(A)** Shows bifidobacterial extracellular structures-mediated interaction with the host immune system. Pili are depicted as black appendages (Pili+) while exopolysaccharides structures (EPS+) are displayed as light blue layers around the cells. Blue bifidobacterial shapes correspond to bifidobacterial strains. **(B)** Exhibits the cross-feeding effects between species of the *Bifidobacterium* genus and butyrate-producing bacteria. Acetate and lactate produced by bifidobacterial species by degrading mucin, HMO or diet-derived glycans become carbon sources for butyrate-producing microorganisms, stimulating a butyrogenic effect. At the bottom of the image, host immune system cells are represented.

Microorganisms have evolved specific strategies to interact with the host. Despite the health-promoting effects exerted by bifidobacteria, the molecular mechanisms exploited by these microorganisms to colonize the gut, adhere to the host intestinal epithelium and elicit an immune response are still largely unknown. In this context, certain extracellular structures, excreted enzymes and/or bioactive metabolites seem to play a pivotal role in host-microbe interaction, thereby modulating the immune system. In the following sections, key examples of these extracellular structures will be discussed in detail (Figure 1A; Table 1).

**Table 1:** Bifidobacterial extracellular molecules and/or metabolites exerting an immunological effect.

**Strains**	**Molecules**	**Target**	**Effect**	**References**
*B. bifidum* PRL2010	Sortase-dependent pili	Macrophages	Induction of high level of TNF-α and reduced expression of IL-12	(48)
	Bacteria-like particles exposing PRL2010 sortase-dependent pili major subunit	TNBS-induced colitic mice	Reduction of colitis symptoms	(49)
*B. breve* UCC2003	Tad pili	Epithelial cells	Stimulation of the intestinal mucosa/mucosal homeostasis	(50)
	EPS	Mice splenocytes	Reduction of proinflammatory cytokines (IFN-α, TNF-α, and IL-12)	(51)
		*Escherichia coli* 0111:B4 lipopolysaccharides-injected mice	Reduction of apoptotic epithelial cell shedding and inflammatory response	(52)
*B. animalis* subsp. *lactis* A1dOxR	EPS	PBMCs	Lower release of inflammatory cytokines	(53)
*B. adolescentis* IF1-03	EPS	RAW264.7 macrophages or mice splenocytes	Production of anti-inflammatory cytokines (IL-10, IL-6, and TGF-β) and increased proportion of the T_reg_ cells	(54)
		DSS-induced colitis mice	Induction of high levels of anti-inflammatory cytokines, reduction of the ulceration area and thickening of the intestinal wall	(54)
*B. longum* subsp. *longum* YS108R	Ropy-EPS	DSS-induced colitis mice	Reduction of colonic injury, myeloperoxidase activity and inflammatory cell infiltrations and preservation of the crypt structures from damage. Reduction of IL-6 and IL-17 and accumulation of the anti-inflammatory cytokine IL-10	(55)
*B. longum* subsp. *longum* 35624	EPS	T cell transfer colitis model mice	Prevention of colitis related symptoms	(56)
		Ovalbumin respiratory allergy model mice	Enhanced recruitment of IL-17^+^ lymphocytes to the lung	(57)
			Suppression of Th2 type immune response in lungs	(57)
*B. longum* subsp. *longum* NCC2705	Serpin	Peptic-tryptic digestion of gliadin-sensitized mice	Protection against gliadin-induced immunopathology	(58)
	Acetate (produced by diet-derived glycans degradation)	*E. rectale* ATCC 33656	Butyrogenic and bifidogenic effects	(59)
*B. bifidum* MIMBb75	TagA	Human DCs	Activation/proliferation of DCs and induction of IL-2	(60)
	BopA	Caco-2 cells	Probably involved in stimulating IL-8 production	(61)
*B. adolescentis* L2-32	Acetate (produced by diet-derived glycans degradation)	*F. prausnitzii* S3L/3	Butyrogenic effect	(62)
		*F. prausnitzii* A2-165	Butyrogenic effect	(62)
*B. bifidum* strains	Acetate (produced by mucin degradation)	*E. hallii* DSM 3353	Butyrate and propionate production	(63)
*B. longum* subsp. *infantis*	Acetate (produced by HMO degradation)	*E. hallii* DSM 3353	Butyrate, propionate, and formate production	(64)

### Pili

Pili (or fimbriae) are described as long proteinaceous appendages produced by bacteria, protruding from the extracellular cell surface, and that may be involved in microbe-host interactions by promoting adhesion to the intestinal epithelium or facilitating aggregation with other bacterial cells (65–67). In bifidobacteria, two different types of pili have been described, i.e., the sortase-dependent pili and the type IVb pili, which are also known as tight adherence pili (Tad pili) (68, 69).

To support the notion that these fibers play a role in host-microbe interaction, it was demonstrated that bifidobacterial sortase-dependent pili biosynthesis is only activated under particular conditions, such as during *in vivo* colonization, *in vitro* contact between bifidobacterial cells and human cell lines, and/or exposure to certain extracellular matrix proteins (48, 68–70). Genes encoding the biosynthetic enzymes for a particular sortase-dependent pilus are generally clustered together in a genetic locus, encompassing a gene encoding a major pilin protein, as well as one or two genes involved in the synthesis of (one or two) ancillary pilins and a third gene implicated in the expression of a pilus-specific sortase (66, 71). The sortase is a transpeptidase able to cross-link the pilus building blocks and covalently connect the resulting polymer to the cell wall surface (71). Comparison of the sequenced genomes from different bifidobacterial species revealed the presence of various sortase-dependent pilus-encoding loci in terms of number and genetic sequence variability (68). Indeed, the number of these pilus-encoding loci was observed to vary from a total absence typical of some *Bifidobacterium actinocoloniforme, B. longum* subsp. *longum*, and *B. longum* subsp. *infantis* strains to up to seven pilus-encoding loci found in the genome of *B. dentium* Bd1 (66, 68). Moreover, the GC content deviation and different codon usage observed in bifidobacterial sortase-dependent loci together with their frequent localization near transposons indicate that these genetic elements have been acquired through horizontal gene transfer events (68, 72).

Mediating adhesion and interaction with the host is not the only role exerted by bifidobacterial sortase-dependent pili. Indeed, they are also implicated in microbe-microbe interactions and in stimulating/modulating the host immune system. In this context, a case study focused on *B. bifidum* PRL2010 demonstrated that sortase-dependent pili have a pioneering role in favoring the aggregation between bacterial cells of a heterogeneous population, enhancing the colonization of host intestinal mucosa (70). In the same way, a related study highlighted that sortase-dependent pili produced by *B. bifidum* PRL2010 can activate various signals in macrophages inducing local high levels of TNF-α cytokines, but a reduced expression of other proinflammatory cytokines, such as IL-12, associated with systemic response (48), thus favoring initial cross-talk between this bifidobacterial strain and host immune cells without causing a detrimental inflammatory cascade response. In this context, as bifidobacteria are among the first colonizers of the human gut (29) and considering that the immune system of newborns is largely immature, the proinflammatory stimuli provoked by pili of *B. bifidum* PRL2010 (and possibly other infant-associated bifidobacteria) may be an essential starting point to prime the immune system (48). To further emphasize the immune-modulatory features exerted by *B. bifidum* PRL2010 pili, an *in vivo* study based on the administration of bacteria-like particles (BLP-FimP_PRL2010_) that expose the *B. bifidum* PRL2010 sortase-dependent pili major subunit 3 days before the treatment of mice model with 2,4,6-trinitrobenzensulfonic acid (TNBS) to induce colitis, clearly showed a reduction of biological/clinical markers associated with colitis symptoms when compared to the control. In addition, administration of BLP-FimP_PRL2010_ causes TNF-α overexpression coupled with a significantly reduction of IL-10 response in TNBS-induced colitic mice. Thus, alerting the immune system and enhancing the host reaction to inflammation, which is a typical condition of colitis (49).

Another pilus type produced by bifidobacterial species are the so-called Tight ADherence (Tad) pili. These structures were first described in the pathogenic Gram-negative *Actinobacillus actinomycetemcomitans*, where these pili were shown to mediate adhesion to the host cell surface, supporting colonization and pathogenesis through the formation of biofilms (73, 74). The gene cluster responsible for the biogenesis of Tad pili encodes an ATPase, two transmembrane proteins and a septum site-determining protein that together constitute the pilus production, secretion, and positioning apparatus in addition to a peptidase able to post-translationally cleave the hydrophobic leader peptide of prepilins and pseudopilins, the precursors of Tad pili structural proteins (74). The Tad pilus-encoding gene cluster is highly conserved in bifidobacterial genomes (75), although this pilus type has only been characterized in detail in *B. breve* UCC2003 (50, 68, 69). Transcriptomic and gene mutagenesis experiments revealed that *B. breve* UCC2003 *tad* locus are expressed only *in vivo* conditions emphasizing its fundamental role in colonization and persistent adhesion to the host intestinal cells (69, 74). Moreover, in a recent study, the capability of the *B. breve* UCC2003 Tad pili to promote *in vivo* colonic epithelial proliferation was observed 5 days post-administration of the strain to murine model (50). Further analyses, exploiting advanced functional genomic approaches demonstrated, both under *in vivo* and *in vitro* conditions, that the epithelial proliferation response is specifically provoked by the TadE protein. Therefore, bifidobacterial Tad pili may contribute to the maturation of epithelial cells of newborns, stimulating growth of their thin intestinal mucosa and contributing to host mucosal homeostasis (50).

### Extracellular Polysaccharides

The envelope of a wide range of bacteria is surrounded by one or more glycan layers known as capsular polysaccharides (CPS) or exopolysaccharides (EPS). Based on their monosaccharide composition and biosynthesis mechanism mediated by glycosidic linkages, bacterial CPS/EPS (NB. since it is hard to distinguish these, we will refer to these polysaccharides as EPS for ease) can either be classified as homopolysaccharides, when composed by the repetition of a single type of monosaccharide, or as heteropolysaccharides when formed by two or more types of monosaccharide subunits (76). *In silico* analyses performed on bifidobacterial sequenced genomes showed the lack of a “consensus” functional-structural organization in the *eps*-encoding clusters, highlighting consistent inter/intra-species variability in terms of both length and number of genes (77, 78). The scientific community has in recent years increased its interest in EPS producers as these extracellular polymers have been reported to exert a crucial role in human health by promoting adhesion to the intestinal mucosa, as well as by modulating the intestinal microbiota composition, and conferring selective advantage to bacteria through protection to adverse conditions such as presence of bile salts or pH insults (51, 79). In addition, some of these microbial biopolymers are receiving renewed interest due to their involvement in promoting human health (51, 78, 79). In this regard, an *in vitro* experiment aimed at evaluating the level of pro- and anti-inflammatory cytokines stimulation when EPS purified from 10 bifidobacterial strains were co-cultivated with human peripheral blood mononuclear cells (PBMC) revealed that the differentiation of T cells toward T-helper(Th)1 (IL-12/IL-10), Th2 (IL-10/TNF-α), and Th17 (IL-1β/IL-12) effector cells is highly influenced by the physical-chemical features of the particular EPS used in the experiment. This finding suggests that a positive correlation exists between the composition, structure and size of a given EPS polymer and the corresponding elicited immune response (80). In support of this discovery, relevant results were obtained by a further *in vitro* study where PBMCs were co-incubated with the EPS purified from three isogenic *B. animalis* subsp. *lactis* strains, i.e., A1, A1dOx, and A1dOxR strains. The three EPS polymers differ in their molecular mass with the highest molecular weight held by the A1dOxR EPS. Analysis of cytokine profiles revealed that the A1dOxR extracellular polymer induces the lower release of both pro- and anti-inflammatory cytokine by PBMCs when compared to other bifidobacterial strains (53, 81). Similar results were obtained when the immunoregulatory effects elicited by two *B. adolescentis* strains (IF1-03 and IF1-11) were evaluated. In detail, the two *B. adolescentis* strains differ in the exopolysaccharide-mediated enterocyte adhesion/aggregation phenotypes, with *B. adolescentis* IF1-03 possessing the high-molecular-weight EPS. Specifically, *in vitro* co-culturing experiments of the two *B. adolescentis* strains with RAW264.7 macrophages or mice splenocytes showed that while strain IF1-03 stimulated production of anti-inflammatory cytokines such as IL-10, IL-6, and TGF-β, it also increased the proportion of T-regulatory (T_reg_) cells. Notably, *B. adolescentis* IF1-11 induced higher levels of pro-inflammatory cytokines influencing the CD4^+^ T cells differentiation into Th17 cells. This observation was further confirmed by *in vivo* administration of the two above mentioned strains individually to Dextran Sodium Sulfate (DSS)-induced colitis mice. In this case, *B. adolescentis* IF1-03 not only induced high levels of anti-inflammatory cytokines, but it was also shown to contribute to the reduction of the area of ulceration and thickening of the intestinal wall (54). In the same manner, a recent study demonstrated differential alleviative effects in DSS-induced colitis mice when they were individually treated with three different *B. longum* subsp. *longum* strains, i.e., HAN4-25, which is unable to produce EPS, C11A10B producing non-ropy EPS and the producing ropy-EPS strain YS108R, obtained as a spontaneous single nucleic acid mutant from C11A10B. Particularly, *B. longum* subsp. *longum* YS108R reduced the colitis-induced colonic injury, decreasing the myeloperoxidase activity, preserving the crypt structures from damage and moderating the inflammatory cell infiltrations. Moreover, the administration of YS108R correlated with a reduced IL-6 and IL-17A concentration in parallel with an increased accumulation of the anti-inflammatory cytokine IL-10 in the serum coupled with lower IL-6 colonic expression level (55). All together these finding corroborates the notion that EPS-mediated immune response is largely influenced by the physicochemical nature of these polymers (53, 54, 81).

Moreover, similar results were observed when splenocytes isolated from naïve mice were co-cultivated with the EPS-producing *B. breve* UCC2003 strain (EPS^+^) or the two EPS-deficient *B. breve* UCC2003 strains (EPS^−^) obtained by means of an insertion or deletion in the *eps* locus of this bifidobacterial strain. In this regard, splenocytes stimulated with EPS^+^ strain evoked lower expression of proinflammatory cytokines IFN-α, TNF-α, and IL-12 compared to the EPS^−^. This finding was further confirmed through analysis of intracellular cytokine expression in splenocytes isolated from naïve mice orally fed with EPS^+^ and EPS^−^ strains, evidencing significantly altered cytokine profiles between the two experimental conditions. Specifically, a lower proinflammatory cytokine induction was observed in both adaptive and innate immune response cell populations in case of EPS^+^ administration. Altogether these findings suggest that the *B. breve* UCC2003 EPS layer plays a crucial role in the persistence of the strain in the host intestine, reducing the risk of immune clearance against this microbial strain (51). In addition, *B. breve* UCC2003 EPS was shown to exert beneficial effects by reducing apoptotic epithelial cell shedding, which is a condition that occurs in case of IBD patients, where it causes high levels of apoptotic extrusion of small intestinal epithelial cells (IECs) from villi (52). It was previously described that the genome of *B. breve* UCC2003 possesses two adjacent, oppositely orientated gene sets, i.e., *esp1* and *eps2*. The expression of these two transcriptional units, involved in the production of two distinct EPS (EPS1 and EPS2), depends on the orientation of a single promoter. Therefore, only one of the two types of extracellular polymer can be produced at a given time (82). Based on these observations, *B. breve* UCC2003 EPS-positive, *B. breve* UCC2003 EPSdel (a deletion mutant unable to express neither EPS1 nor EPS2) and *B. breve* UCC2003Inv (able to express only EPS2) were administered to mice model, which were then treated with an intraperitoneal injection of lipopolysaccharides (LPS) from *Escherichia coli* 0111:B4 inducing cell shedding (52). Interestingly, when colonized with *B. breve* UCC2003del or *B. breve* UCC2003Inv, no significant protection against cell shedding was observed when compared to the control PBS (Phosphate Buffered Saline)-gavage mice, thus underscoring the strain variant specificity in exerting protective effects. Moreover, evaluation of transcriptional changes in murine small intestine samples from control (PBS) and colonized groups (*B. breve* UCC2003 EPS-positive and *B. breve* UCC2003del) revealed down-regulation of genes related to the apoptotic cascade in mice treated with *B. breve* UCC2003 EPS-positive bacteria. In this context, the EPS of *B. breve* UCC2003 appears to downregulate inflammatory and apoptotic responses, thus reducing cell shedding events (52). Another bifidobacterial strain exerting beneficial effects is represented by *B. longum* subsp. *longum* 35624. The analysis of its genome revealed the presence of a gene cluster (*eps*_624_) encoding the EPS biosynthetic machinery. Despite the fact that the genome comparative analysis showed that *B. longum* subsp. *longum* 35624 EPS-specifying cluster is in the same location in other *B. longum* subsp. *longum* genomes, a different genetic composition of the *eps*_624_ was observed when compared to other analyzed *B. longum* subsp. *longum* strains (83). Based on these findings, further studies were carried out to explore the role played by the EPS in influencing the host immune response. A first study used *B. longum* subsp. *longum* 35624 strain and its derivatives: the exopolysaccharide-negative mutant (EPS^neg^) with an insertion mutation in the *eps* cluster and the EPS^comp^ where the EPS production was restored by genetic complementation of the EPS^neg^ strain. Co-culture of human PBMCs or monocyte-derived dendritic cells (MDDCs) with one of these strains revealed that increased proinflammatory cytokine secretion is specifically related to the lack of EPS, with a marked increase of IL-17. In the same way, administration of these strains individually to mice of a T cell transfer colitis model underlined the ability of the two EPS-producing strains to prevent disease related symptoms, while EPS^neg^ does not show any protection against the development of colitis, with increased recruitment of IL-17^+^ lymphocytes to the gut (56). Moreover, the intranasal administration of EPS-producing *B. longum* 35624 and the EPS^neg^ strains to ovalbumin respiratory allergy model mice, resulted in enhanced recruitment of IL-17^+^ lymphocytes to the murine lung. In this context, a further study showed the capability of *B. longum* 35624 EPS to suppress the Th2 type immune response within the lungs of ovalbumin sensitized mice when they were intranasally treated with purified *B. longum* 35624 EPS (57). All together these observations emphasize that the EPS of *B. longum* 35624 plays an important role in reducing the proinflammatory response.

### Serpins

Serpins (Serine protease inhibitors) represent a superfamily of proteins acting as eukaryotic-type serine protease inhibitors. These prokaryotic enzymes, synthetized by particular members of the intestinal community, are involved in the regulation of a wide range of protease-mediated processes (84, 85). Analyses of the bifidobacterial genome sequences revealed that the presence of serpin-like gene is not ubiquitous but restricted to *B. breve, B. longum* subsp. *longum, B. longum* subsp. *infantis, B. longum* subsp. *suis, B. cuniculi, Bifidobacterium scardovii*, and *B. dentium* species (86). Moreover, an *in vitro* study underlined that the activation of bifidobacterial serpin-encoding genes is driven by specific serine proteases. Transcriptional profiling experiments carried out on RNA extracted from *B. breve* 210B cells treated with different proteases highlighted that the highest up-regulation of the serpin-encoding locus is reached after exposure to papain (86). Conversely, a different bifidobacterial strain, *B. longum* NCC2705, synthetizes a serpin able to act as an efficient inhibitor of human neutrophil elastase (HNE) and, to a lesser extent, pancreatic elastase (87). Considering that the release of serine proteases may occur following intestinal inflammation caused by bacterial infection or intestinal tissue damage typical of inflammatory bowel diseases or ulcerative colitis, the production of serpins may elicit anti-inflammatory activity counteracting the negative effects of (high levels of) human serine proteases. Moreover, the self-produced serpins may assist bifidobacteria to protect themselves against host-derived proteases and survive in a competitive environment (86, 88). To further emphasize the anti-inflammatory role of these prokaryotic enzymes, a recent *in vivo* study demonstrated the efficacy of a specific *B. longum* NCC2705 serpin in the prevention of gluten-related immunopathology. Administration of the wild-type *B*. *longum* NCC2705 (*srp*^+^) to sensitized NOD/DQ8 mice with peptic-tryptic digestion of gliadin, which is one of the main gluten protein fractions, favored protection against the development of gliadin-induced immunopathology when compared with the administration of the knockout *B. longum* NCC2705 (*srp*^−^) that is unable to synthetize serpins. Because the aberrant effects of gluten-related immunopathology are strongly relieved in mice treated with the wild-type strain, it can be speculated that serpins are involved in immune regulation, maintenance of barrier function and inhibition of elastases released during inflammation (58).

### Other Extracellular Molecules Involved in the Bifidobacterial-Host Immune System Interaction

In addition to pili, EPS and serpins, other bifidobacterial-associated extracellular proteins have been implicated in impacting on the host immune system. For instance, TagA is a protein expressed on the outer surface of *B. bifidum* MIMBb75 formed by two active domains: a lytic murein transglycosylase (LT) and a cysteine- and histidine-dependent amidohydrolase/peptidase (CHAP). This protein acts as a peptidoglycan lytic enzyme that when co-cultured with human DCs was shown to cause activation/proliferation of the DCs itself and the induction of IL-2 (60). *B. bifidum* MIMBb75 also produces the surface-associated protein named BopA, which has been reported to not only enhance adhesion of *B. bifidum* MIMBb75 to Caco-2 cell lines, but also to stimulate production of IL-8 by Caco-2 cells (61). However, subsequently the role of BopA in mediating adherence of *B. bifidum* to epithelial cells has been re-evaluated. In this setting, the gene encoding BopA was cloned and expressed in *E. coli* without the BopA-associated lipobox and hydrophobic signal peptide, resulting in a reduced capability of BopA to adhere to Caco-2 and HT-29 cells. Previous results may therefore have been compromised by an unspecific effect caused by higher hydrophobicity of the BopA (lipo)proteins (89).

## Bifidobacterial Metabolism Influences Intestinal Immune Homeostasis and Inflammatory Response Through a Microbe-Microbe Cross-Feeding Activities

The ability to degrade complex carbohydrates that cannot be metabolized by the host is one of the key genetic features of bifidobacteria (90, 91). The nondigestible carbohydrates degraded by these commensal microorganisms include diet-derived sugars (e.g., glucans, xylans, pectins, fructans, cellulose, resistant starch) as well as host-derived glycans, encompassing Human Milk Oligosacchardes (HMO) and mucins, which are glycoproteins covering the gut epithelium and secreted by the goblet cells of the GIT, in the form of *O*-linked and/or *N*-linked glycans (90). The genetic arsenal required for the utilization of the aforementioned compounds is generally organized in gene clusters including glycosyl hydrolases (GH)-encoding genes coupled with gene sequences responsible for the assembly of sugar-specific ATP-binding cassette (ABC) transporters, permeases, proton symporters, and phosphoenolpyruvate-phosphotransferase systems (72, 90). Specifically, the degradation of complex carbohydrates requires dedicated extracellular and/or intracellular GHs to generate mono- and/or oligo-saccharides, which are then internalized by the transport systems. After reaching the cytoplasm, those carbohydrates that still need to be processed may be subject to further modification such as deacetylation, deamination, epimerization, and phosphorylation to produce phosphorylated monosaccharides before entering the final central metabolic route, known as the “bifid shunt,” where simple and complex sugars eventually converge for energy production (92). At the end of this specific pathway, 2.5 ATP molecules per Mol of glucose are theoretically obtained together with 1 Mol of lactate and 1.5 Mol of acetate. Particular interest has focused on the production of Short Chain Fatty Acids (SCFAs) by the intestinal microbiota as they contribute to the host health through increasing calcium and magnesium absorption, regulating bowel functions, providing nutritional support to colonocytes, decreasing the luminal pH and thus preventing overgrowth of pH-sensitive pathogenic bacteria and finally influencing the host immune system (93, 94). Structurally defined as saturated aliphatic organic acids, major SCFAs at the site of intestine are represented by acetate, propionate and butyrate. Among the SCFAs produced in the human colon, butyrate displays a key role in the gut health stimulating the development of the intestinal cells. Indeed, being the preferred energy source for intestinal epithelial cells, butyrate consumption improves host IEC integrity promoting tight junctions, cell proliferation, and mucin production by Goblet cells (93, 95). Moreover, butyrate is involved in guiding the immune system toward an anti-inflammatory response (93, 96, 97). For instance, butyrate exhibits anti-inflammatory properties inducing the production of TGF-β, IL-18, and IL-10 cytokines by both antigen presenting cells and IECs coupled with the stimulation of the differentiation of naïve T cells to Treg cells (98, 99) (Figure 1B).

Although bifidobacteria are unable of directly synthetizing butyrate, as acetate-producing microorganisms, they may indirectly influence the activity and composition of the butyrogenic members of the gut microbiota and the immune response through a mutual beneficial cross-feeding interaction. Indeed, bifidobacteria can operate as primary players degrading complex carbohydrates to produce acetate which is then consumed by secondary degraders that generate butyrate, thus stimulating a butyrogenic effect. Most of butyrate producers in the human colon belong to the Firmicutes phylum, specifically to the clostridial clusters IV and XIVa encompassing *Faecalibacterium prausnitzii, Eubacterium rectale, Eubacterium hallii, Roseburia* spp. (*Roseburia hominis, Roseburia intestinalis*, and *Roseburia inulinivorans*) and *Anaerostipes* spp. (*Anaerostipes caccae, Anaerostipes hadrus*, and *Anaerostipes butyraticus*) (92, 100). In this context, several studies have reported on the butyrogenic effects resulting from the degradation of diet-associated carbohydrates by bifidobacteria (Table 1). Particularly, the co-culture of *B. longum* subsp. *longum* NCC2705 and *E. rectale* ATCC 33656 in a growth medium with arabinoxylan oligosaccharides (AXOS) as unique carbon source affected the growth yield of both strains when co-cultivated compared to the respective mono-cultures, leading to a mutual beneficial effect. Notably, *B. longum* NCC2705 as an arabinose substituent degrader of AXOS, possesses the AXOS-degrading enzymes whose action allow the release of xylose backbone (XOS) and acetate. On the other side, *E. rectale* ATCC 33656 consumes the whole (A)XOS substrate releasing xylose and arabinose. In this way, *E. rectale* ATCC 33656 takes advantage of acetate for butyrate production, resulting in a butyrogenic effect, while *B. longum* NCC 2705 consumes (A)XOS monosaccharides, leading to an increase in acetate production and ensuring its cell concentration thus stimulating a bifidogenic effect (59). Similar findings were obtained by the co-cultivation of *F. prausnitzii* S3L/3 and *B. adolescentis* L2-32 in the presence of fructooligosaccharides (FOS 95), or the co-cultivation of *F. prausnitzii* A2-165 and *B. adolescentis* L2-32 on starch, resulting in both cases in an enhanced production of butyrate when compared to the mono-cultures (62).

As mentioned above, bifidobacteria can produce acetate not only through the consumption of diet-derived carbohydrates, but also by degrading host-derived glycans. In this regard, it has been demonstrated that a cross-feeding interaction is established when *B. bifidum* strains are singularly co-cultured with *E. hallii* DSM 3353 in presence of mucin as the only carbon source (63). Gut colonization by *E. hallii* generally occurs in the first months after birth reaching adult abundance levels between 5 and 10 years of age (64). This microorganism is able to consume acetate and lactate to produce butyrate and propionate, thus promoting a beneficial effect on gut health during childhood. However, *E. hallii* does not possess genes involved in the degradation of complex host- or diet-derived glycans, relying on other microbial players for the supply of simpler carbohydrates (101). Conversely, *B. bifidum* is one of the prominent bifidobacterial species able to utilize mucin as carbon source thanks to the presence in their genome of genes encoding for α-fucosidases, α-sialidases, endo-α-N-acetylgalactosaminidases, and N-acetyl-β-hexosaminidases (102). The ability of *B. bifidum* to degrade mucin into mucin-associated mono- and oligo-saccharides coupled with the production of acetate and lactate stimulate *E. hallii* colonization and consumption of simple glycans released by *B. bifidum* as energy source and the acetate and lactate for butyrate production (63). Moreover, three-strain fermentations involving *B. bifidum* and *E. hallii* with *B. breve* or *B. longum* subsp. *infantis* on mucin as the sole carbon source showed the formation of propionate in both three-strain co-cultures by *E. hallii*, emphasizing the health-promoting effect of cross-feeding interactions as propionate acts as a precursor for gluconeogenesis in the liver and affects intestinal homeostasis with anti-inflammatory and anti-carcinogenic actions (63, 103). A similar trophic interaction was observed when the same strain, *E. hallii* DSM 3353, was co-cultured with *B. longum* subsp. *infantis* strains in presence of fucosyllactose (FL; either 2′- or 3-FL), which is a component of the HMO. *E. hallii* does not grow on HMOs as the unique carbon source. In contrast, *B. longum* subsp. *infantis* utilizes the lactose moiety of FL to produce lactate and acetate and metabolize L-fucose to 1,2-propanediol (1,2-PD). These by-products are then used by *E. hallii* for its growth and the formation of butyrate coupled with propionate and formate (64).

Overall, these observations strengthen the notion that bifidobacterial abilities to degrade non-digestible diet- and/or host-derived glycans coupled with subsequent production of acetate (or 1,2-PD) have an indirect role in the modulation of the host immune system, stimulating the growth and production of butyric acid by butyrate-producing intestinal microorganisms.

## Conclusions

In recent decades, a growing number of studies have demonstrated that changes in the intestinal microbial composition and gut homeostasis is directly linked to intestinal disorders resulting, in the most severe cases, in IBD and colorectal cancer. The modulation of the gut microbiota through prebiotics and/or probiotics is one of the ways to counteract these dysbiosis, improving human health. In this context, bifidobacteria are generally considered as potent probiotics for their health-promoting features. Indeed, they trigger immunomodulatory responses aimed at maintaining the host intestinal homeostasis through different mechanisms such as production of extracellular structures that can interact with other intestinal microorganisms and/or the host cells coupled with the release of by-products of their metabolism that may be utilized by other commensal bacteria, such as the butyrate-producing microorganisms, to establish a cross-feeding interaction. However, despite their key role in stimulating human health and development in technology, the precise mechanism by which bifidobacteria solicit an immune response is far from being fully understood. Indeed, most studies intended to correlate bifidobacteria and their immune modulatory effects have been carried out by exploiting cell lines and/or animal models, thus preventing a comprehensive understanding of the impact that bifidobacteria may exert on the human immune system. Indeed, because of the complexity of the gut microbiota, in case of *in vivo* studies it is possible that the presence of other bacterial strains may influence or even reverse the bifidobacterial immunomodulatory effects. Therefore, the challenge for future studies is to deepen the knowledge about this field, overcoming the abovementioned limits.

## Author Contributions

All authors listed have made a substantial, direct and intellectual contribution to the work, and approved it for publication.

### Conflict of Interest

The authors declare that the research was conducted in the absence of any commercial or financial relationships that could be construed as a potential conflict of interest.
